# Supplementary material for: Hardware functional obfuscation with ferroelectric active interconnects
Source: Nat Commun. 2022 Apr 25;13:2235. doi: 10.1038/s41467-022-29795-3 (PMC9038742; doi:10.1038/s41467-022-29795-3)
Supplement: Supplementary file 2 — Supplementary Information [file 41467_2022_29795_MOESM2_ESM.pdf]

Supplementary Materials

Other Important Applications of FeFET Active Interconnect

The variants of our ultra-compact FeFET active inter-connect design can be extended to apply in various chip design applications. Three potential applications that can be used in IC designs are listed in Fig. S1. An example is the design of a configurable path connector capable of connecting/disconnecting inputs to destination units. This is especially beneficial for controlling the logic signal flow towards redundant computation units. Inclusion of redundant functional units is a common method to develop reliable fault tolerant systems. In this application, active ferroelectric based pass transistors can be utilized as path connectors and such units can be used to control the path connectivity between different functional units with ease.

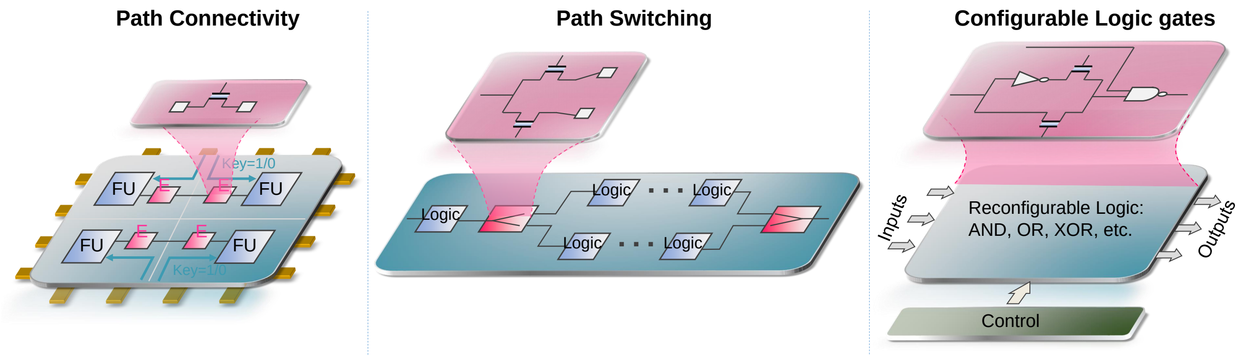

Figure S1: Other important applications that can benefit from ultra-compact FeFET active interconnect. It can be used as path connector, reconfigurable route switching, and reconfigurable logic.

Another potential application is configurable path switching which can essentially act as a router. Multiple active interconnect based pass transistors will be able route/block signals to different functional units as shown in Fig. S1. In addition, active interconnect blocks can also be used to construct reconfigurable logic gates by dynamically programming their control inputs.

Many combinations such as such as NAND, AND, OR, NOR, XOR, XNOR etc are possible by the appropriate design (Fig. S1).

SPICE Simulations and Waveform Analysis

Dynamic programming simulation of the proposed active inter-connect based encryption block is shown in Fig. S2. Spectre is used simulation verification and the schematic of the encryption unit is given in Fig. 2(e). Table. S1 shows simulation parameters. Simulations are carried out using NCSU FreePDK 45 nm technology[57]. FeFET uses verilog-A model to capture its characteristics. In this analysis, a programming pulse (V_P $\pm$4 V) with a pulsewidth of 500 ns is used to set the threshold states of two FeFET pass transistors.

Technology	45 nm
Width	90 nm
Thickness of the ferroelectric	8 nm
Number of domains	20
Supply Voltage	0.8 V
CNTL	1.1 V
V_p	± 4 V, pulsewidth = 500 ns
INPUT	0.8 V

Table S1: Simulation Parameters

First, the block is programmed for buffer mode of operation. Second, the device is reprogrammed for inverter mode of operation. In the buffer mode of encryption, T1 (Fig. 2(f)) is programmed to HVT and T2 is programmed to LVT by asserting the write voltages in CNTL terminals. During this period, the polarizations of these two FeFETs are set in opposite directions. In the evaluation mode, CNTL1 (V_{CNTL1}) and CNTL2 (V_{CNTL2}) are asserted with 1.1V and output (Out) follows the input (In). This is shown as Programming stage/Buffer in Fig. S2.

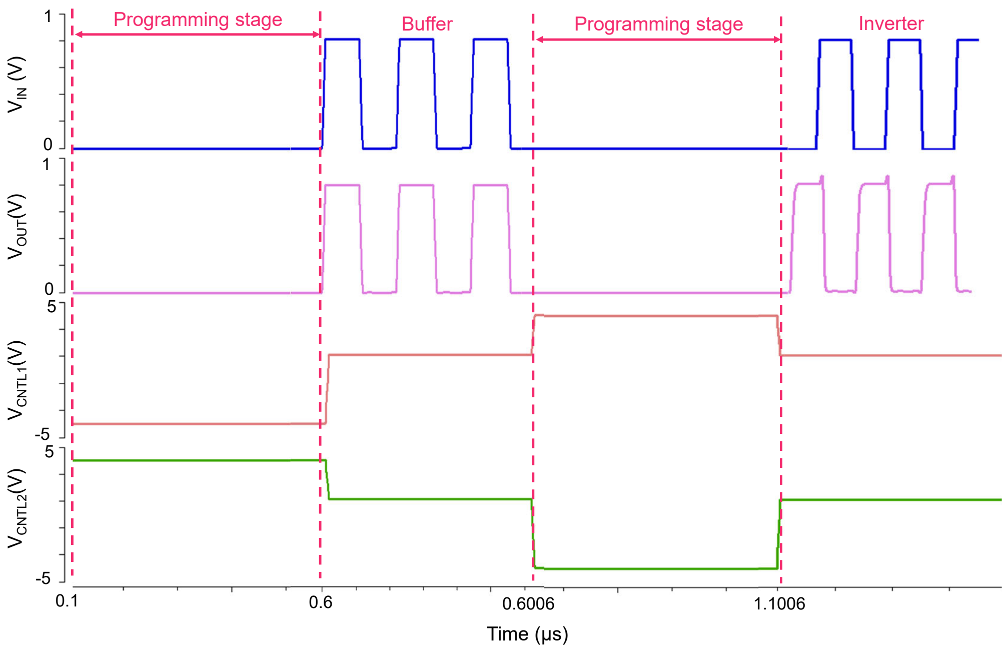

Figure S2: Simulated waveforms of the dynamic programming of the FeFET active inter-connect encryption block (Fig. 2(e)). In the buffer mode programming stage, T1/ T2 is programmed to HVT/ LVT respectively by asserting write voltage on CNTL signals. In the logic mode, read voltage is asserted on CNTL signals and V_{OUT} follows V_{IN}. In the inverter mode programming stage, T1/T2 is programmed to LVT/HVT respectively by asserting write voltage on CNTL signals. In the logic mode, read voltage is asserted on CNTL signals and V_{OUT} shows inverted V_{IN}.

In the inverter mode of encryption, T1 (Fig. 1(h)) is programmed to LVT and T2 is programmed to HVT by asserting the write voltages in CNTL terminals. In the logic mode, CNTL1 (V_{CNTL1}) and CNTL2 (V_{CNTL2}) are asserted with 1.1V and logic input (V_{IN}) is set at 0.8 V for logic high and 0 V for logic low. The output (Out) shows the inverted input (In). In short, the proposed encryption circuit can produce two different outputs from the same input based on FeFETs' programmed states making a strong case for reverse engineering resilient hardware.

Variation Analysis

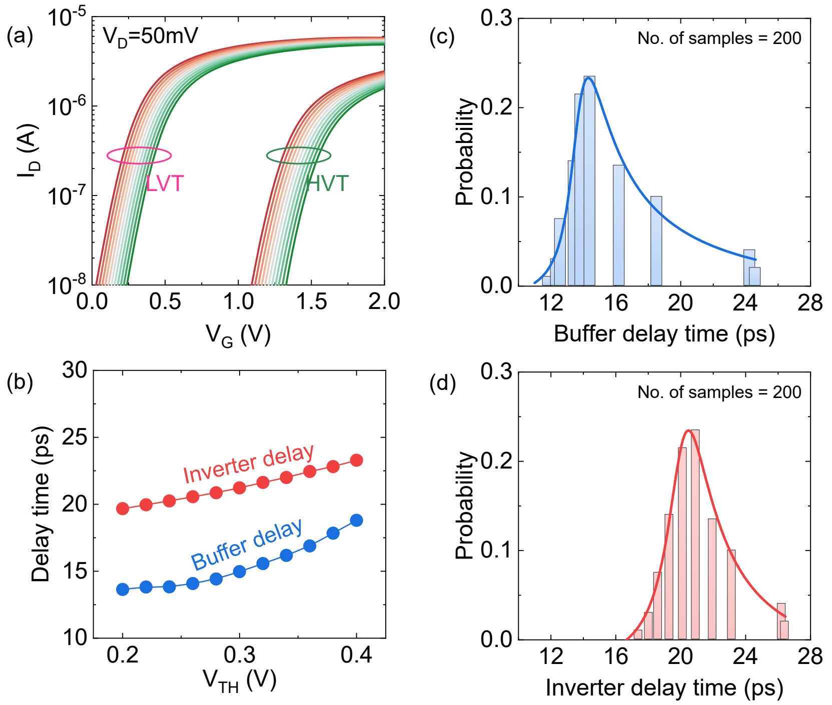

Figure S3: Threshold voltage and delay variation analysis of the FeFET active inter-connect encryption block. (a) Equivalent NMOS I-V characteristics of the FeFET in LVT and HVT states. (b) Delay of the proposed reconfigurable encryption circuit with equivalent nmos transistors in buffer mode and inverter mode. (c) Probability distribution of the delay in reconfigurable encryption circuit with V_T variation when working as a buffer (number of samples: 200). (d) Probability distribution of the delay in encryption circuit with V_T variation when working as an inverter (number of samples: 200).

Variation analysis is conducted to study the delay impact of our proposed circuit. Fig. S3(a) shows I_d - V_g characteristics of the calibrated NMOS transistor of the equivalent FeFET in LVT and HVT states. Monte Carlo simulations are performed to model threshold voltage gaussian

variation. The delay variation of the encryption circuits in inverter mode and buffer mode with respect to threshold voltage change are shown in Fig. S3(b). The probabilistic distribution of the buffer delay and inverter delay with respect to threshold voltage variation is given in Fig. S3(c),(d). The analysis shows an overall delay variation of 6.4 ns/4.5 ns for the proposed circuit in respective buffer/inverter modes .

Layout & Device Analysis

Fig. S4 shows the layout of one encryption block consisting of two FeFETs and one inverter. The block has 1.35 μm (30F) width and 0.81 μm (18F) height. The area is calculated as 1.09 μm^2. Note, compared to the TVD implementation [17] needing 30 transistors, our implementation needs only 4 transistors while maintaining the camouflaging functionality.

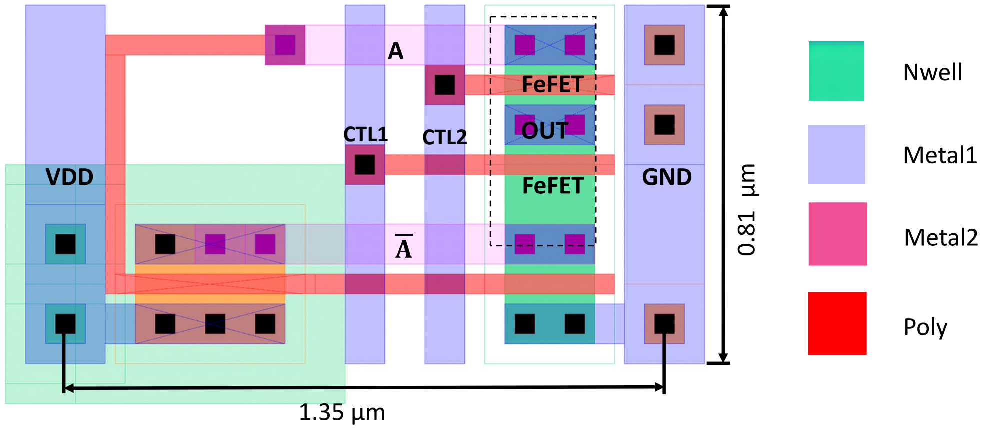

Figure S4: Layout of a single active interconnect based encryption block.

FeFET DC IV Characteristics

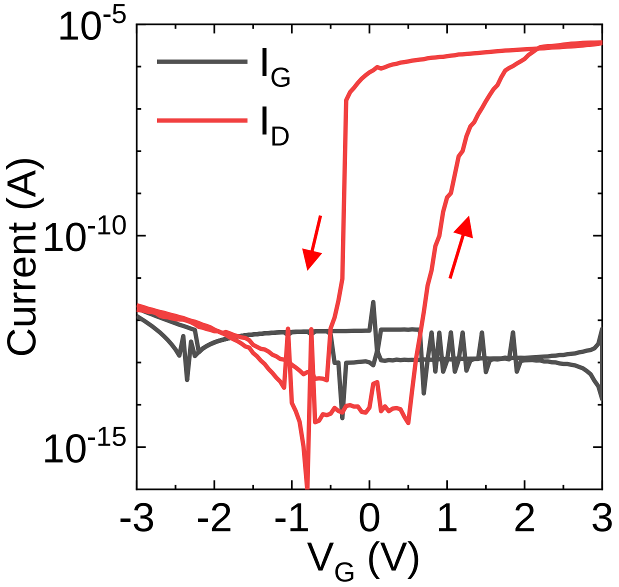

Figure S5: DC Transfer characteristics of a FeFET. I_D-V_G curve shows a large memory window. The I_G-V_G shows a negligible gate current, which is below the noise floor of the instrument, due to a thick ferroelectric layer in the gate stack. This shows that applying a constant evaluation gate bias does not necessarily incur much additional power consumption. Due to its thick gate dielectric, FeFET may actually has a lower gate leakage than normal logic transistor.

FeFET State Stability

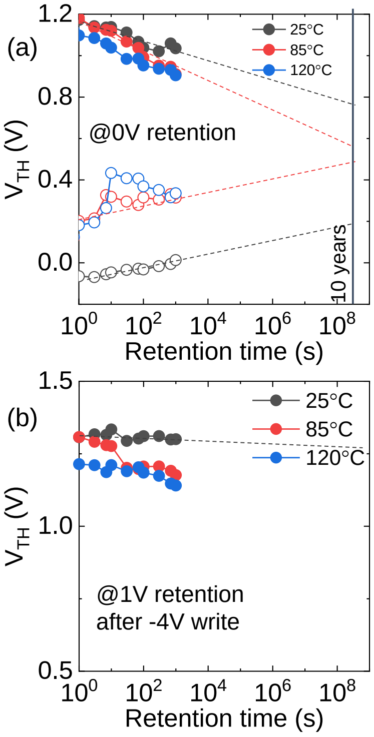

Figure S6: FeFET state stability. (a) Retention characteristics of the LVT and HVT states of FeFET at different temperatures at 0 V retention voltage. (b) Stability of HVT state subjected to +1 V constant stress. Though not the best retention performance reported in FeFET, the device maintains 0.6 V memory window when extrapolated to 10 years at room temperature. In addition, because the evaluation gate bias is constantly applied to FeFET, which might cause concern over the stability of HVT state. Fig. S6(b) clearly suggests that our device could be free from the disturb. Also note that evaluation bias can be shifted close to 0 V by V_{TH} engineering.

INPUT1	INPUT2	NAND<br>(C1=BUF, C2=BUF,C3=BUF)	AND<br>(C1=BUF, C2=BUF,C3=INV)	OR<br>(C1=INV, C2=INV,C3=BUF)	NOR<br>(C1=INV, C2=INV,C3=INV)
0	0	1	0	0	1
0	1	1	0	1	0
1	0	1	0	1	0
1	1	0	1	1	0

Table S2: Truth table of NAND based reconfigurable logic

NAND Gate Based Reconfigurable Logic

Table. S2 gives the configuration of the three inverter-buffer block to configure the NAND based reconfigurable logic to different logic gates.

	FeFET-TVD[19]	ReConfig with 1 AIB	ReConfig with 3 AIB
AND	31T	8T	16T
NAND	31T	8T	16T
OR	31T	N/A	16T
NOR	31T	N/A	16T

Table S3: Number of transistor required for previous FeFET-TVD[17] Circuit and Proposed Reconfigurable logic with active interconnect blocks.

Table. S3 gives a comparison between TVD implementation[17] and reconfigurable logic centred on NAND gate (Tabe. S2) using active interconnect blocks (AIB) based on the number of transistors. Using one active interconnect block at the output of NAND gives reconfigurable AND/NAND logic. The above mentioned circuit takes 8 transistors. In addition, adding 2 more AIBs at the input of NAND give 4 reconfigurable logic possibilities with 16 Transistors.

Double Inversion

In this section, a possibility of a double inversion of data is shown when adding more than one encryption block to the same input-output path. The experiment is conducted by adding multiple active interconnect based encryption blocks to an input-output path in C5315CP. This is done for analysing the impact of double inversion on timing and encryption probability in such paths. A double inversion is defined as two times negation of data in an input-output path. More than one encryption blocks programmed in the inverter mode in a path may lead to double inversion of data with some input data combinations. To demonstrate the logic impact of a double inversion on a final output, a specific path having two encryption blocks from C5315CP is chosen. The screenshot of the timing report of the chosen input-output path is shown in Fig. S7. INVMOD in the timing report is the name of our encryption unit. Fig. S8a shows the schematic of a segment from the chosen path (Fig. S7) before the insertion of encryption units. Fig. S8b shows the same segment of the circuit after inserting one encryption unit in the inverter mode. Fig. S8c shows the circuit segment after inserting two encryption units (both programmed in the inverter mode). It can be observed that for a set of specific inputs, the intermediate output O_{inter} is ”1” as shown in Fig. S8a. After inserting a single encryption unit, O_{inter} value is switched to ”0” as shown in Fig. S8b. After inserting two encryption units, O_{inter} value is switched back to ”1” as shown in Fig. S8c (double inversion at O_{inter}). Fig. S8d shows that changes in intermediate output (O_{inter}) gets transmitted to the final output with certain input combinations. This implies that having more than one encryption units in the same path may leave the original output unchanged in certain conditions decreasing the encryption probability.

```
Report : timing
  -path_type full
  -delay_type max
  -slack_lesser_than 0.00
  -max_paths 200
  -transition_time
  -capacitance
  -sort_by slack
Design : c5315mod
Version: K-2015.12-SP2
Date   : Fri May 28 05:12:35 2021
****************************************


  Startpoint: in[75] (input port)
  Endpoint: out[0] (output port)
  Path Group: (none)
  Path Type: max

  Point                              Cap      Trans      Incr       Path
  ---------------------------------------------------------------------------
  input external delay                                   0.00       0.00 f
  in[75] (in)                        0.02      0.00      0.00       0.00 f
  U1536/Y (MUX2X1)                   0.01      0.09      0.07       0.07 r
  U1535/Y (INVX1)                    0.00      0.01      0.04       0.12 f
  U1078/Y (AND2X1)                   0.01      0.03      0.05       0.17 f
  U1079/Y (INVX1)                    0.00      0.00      0.01       0.18 r
  U1534/Y (OAI21X1)                  0.00      0.01      0.01       0.19 f
  U1533/Y (INVX1)                    0.03      0.12      0.09       0.29 r
  U1044/Y (AND2X1)                   0.01      0.05      0.05       0.33 r
  U1045/Y (INVX1)                    0.00      0.01      0.03       0.36 f
  U1513/Y (OAI21X1)                  0.00      0.05      0.04       0.40 r
  inverter1/Y (INVMOD)               0.01      0.07      0.07       0.48 f
  U1508/Y (AOI21X1)                  0.00      0.02      0.03       0.51 r
  U988/Y (BUFX2)                     0.00      0.01      0.04       0.55 r
  inverter2/Y (INVMOD)               0.00      0.05      0.06       0.61 f
  U1507/Y (XOR2X1)                   0.01      0.09      0.07       0.68 r
  U1502/YS (FAX1)                    0.00      0.01      0.09       0.78 f
  U1501/Y (MUX2X1)                   0.01      0.08      0.07       0.84 r
  U1500/Y (XOR2X1)                   0.00      0.02      0.04       0.89 f
  U1467/Y (AOI22X1)                  0.00      0.04      0.04       0.93 r
  U840/Y (BUFX2)                     0.00      0.01      0.04       0.96 r
  U808/Y (AND2X1)                    0.00      0.03      0.03       0.99 r
  U963/Y (INVX1)                     0.01      0.04      0.04       1.03 f
  U1466/Y (MUX2X1)                   0.00      0.04      0.05       1.08 r
  U1465/Y (MUX2X1)                   0.00      0.01      0.03       1.11 f
  U1464/Y (NAND2X1)                  0.00      0.03      0.02       1.13 r
  out[0] (out)                                 0.03      0.00       1.13 r
  data arrival time                                                 1.13
  ---------------------------------------------------------------------------
  (Path is unconstrained)
```

O_{inter}

Figure S7: Timing report a path from C5315CP

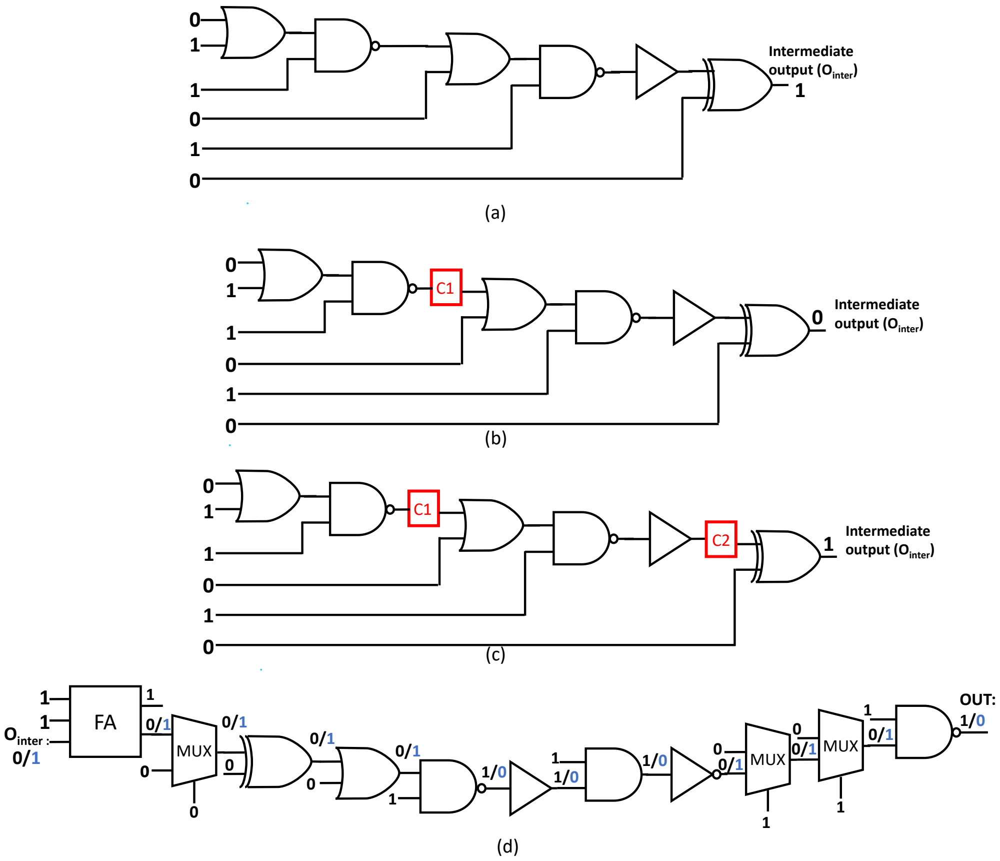

Figure S8: (a) A segment of input-output path. The segment output is named O_{inter}. O_{inter} is "1" with the current set of inputs. (b) Segment in (a) after adding one encryption logic(C1). C1 is programmed in the inverter mode which causes O_{inter} to be "0". (c) Segment in (a) after adding two encryption logic units(C1 & C2). Both C1 & C2 are programmed in the inverter mode which makes O_{inter} to be "1" again. (d) Rest of the schematic from the intermediate output to the selected path's final output. It is seen that changes in intermediate output O_{inter} (due to double inversion) affects the outcome of final output with the given input combinations.

Benchmarking Analysis

Placement Impact

In this section, the impact of the position of placement of the proposed encryption block on encryption probability is analysed. Analysis begins by placing one encryption block at the output of critical path and measuring the encryption probability. Then the encryption block is moved to the input of the current gate and the corresponding encryption probability is recorded. This placement process is repeated till the primary input of the critical path is reached. The experiment results with ISCAS85 benchmarks are shown in Fig. S9. The different levels denote the gate distance from the output. C432 and C499 are small circuits with lesser than 20 levels from the output.

Fig. S9 indicates that for most of the benchmark circuits, encryption probability is at the highest when at level 1. This is where the encryption circuit is placed closest to the output. Then as the encryption element moves away to the center of critical path, encryption probability decreases. This is attributed to the increased potential for logic masking effect with the increase in distance from the output. Also it is observed that, encryption probability further gets increased with further movement towards the input.As the placement moves closer to input, there is an increased potential for higher fanout and more logic branches getting influenced by the encryption logic and hence the potential for altering multiple outputs. In benchmark C5315, encryption probability decreases with level 1, level2, level3 etc. A similar behaviour is observed with C432 as well. C2670 and C1908 show increased encryption probability while moving encryption unit towards the input.

Note, for all encryption and timing analysis in this article, below motioned methodology is adopted. PRIMETIME[56] is used for timing analysis. SPECTRE simulation is used to model the

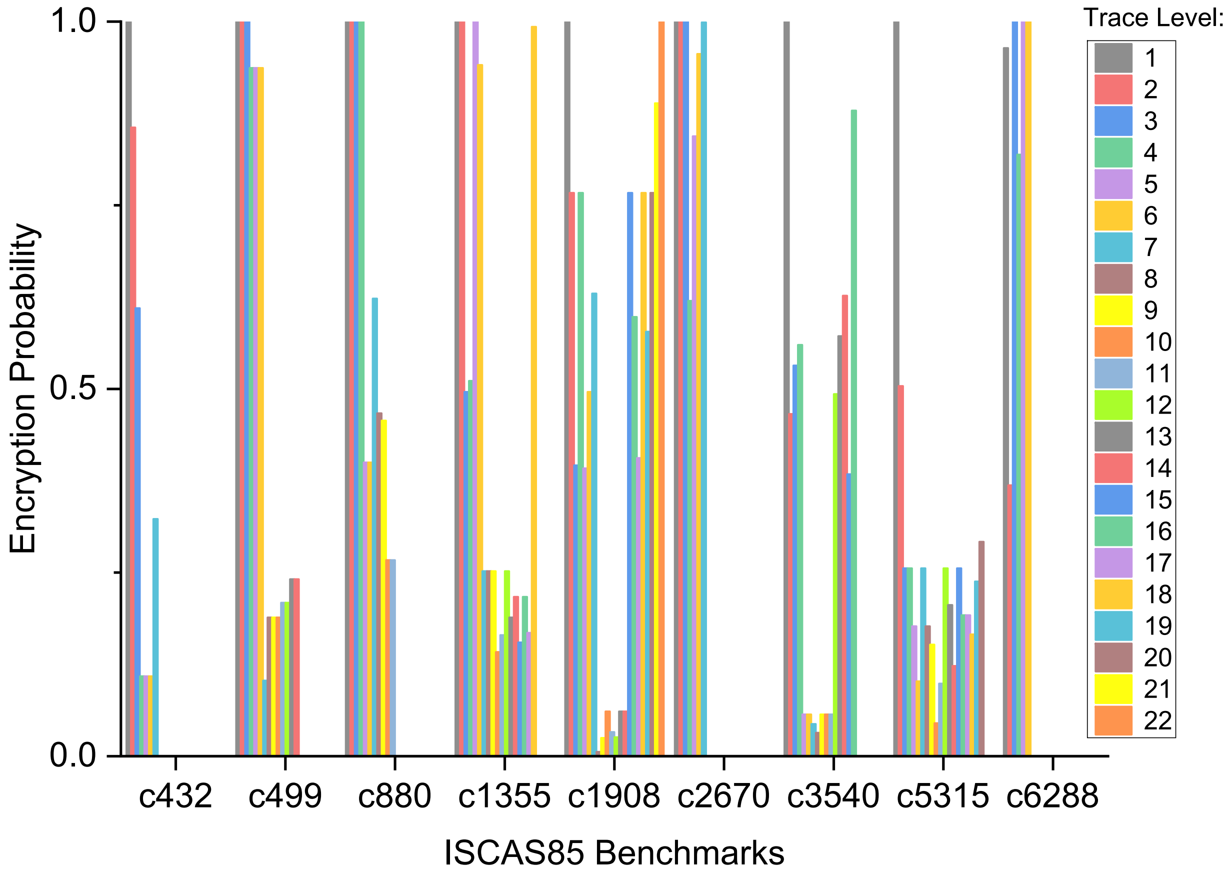

Figure S9: Impact on encryption probability when encryption unit is randomly placed at increasing logic distances from the output pin (number of input test vectors: 1000).

delay of our encryption circuit. Verilog-A is used to capture FeFET behaviour. The simulations are based on NCSU FreePDK 45 nm technology[57]. Functional correctness is verified with Xilinx Vivado [58] on the circuits with encryption blocks for the generated test vectors.In addition these analyses incorporated a timing scaling factor to match the SPICE simulation delay with PRIME TIME library delay . The proposed circuit has used a best case scaling factor of 0.94 and a worst case scaling factor of 2.29 to match the PRIMETIME library delay values.

Peripheral Circuits

A peripheral scheme for encryption key distribution is introduced in Fig. 4. It uses a two step write process to eliminate the negative voltage requirement for logic zero scan output from the flipflop. Here, an alternate design to eliminate two step write process is shown. However the process requires a flipflop where logic zero scan output is biased at a negative voltage. Fig. S10 shows the alternate circuit in programming mode and logic mode.

(a) Mode of operation	L1	L2	L3	L4
Programming	$-V_{write}$	V_{write}	$-V_{write}$	V_{write}
Logic	V_{read}	V_{read}	V_{read}	V_{read}

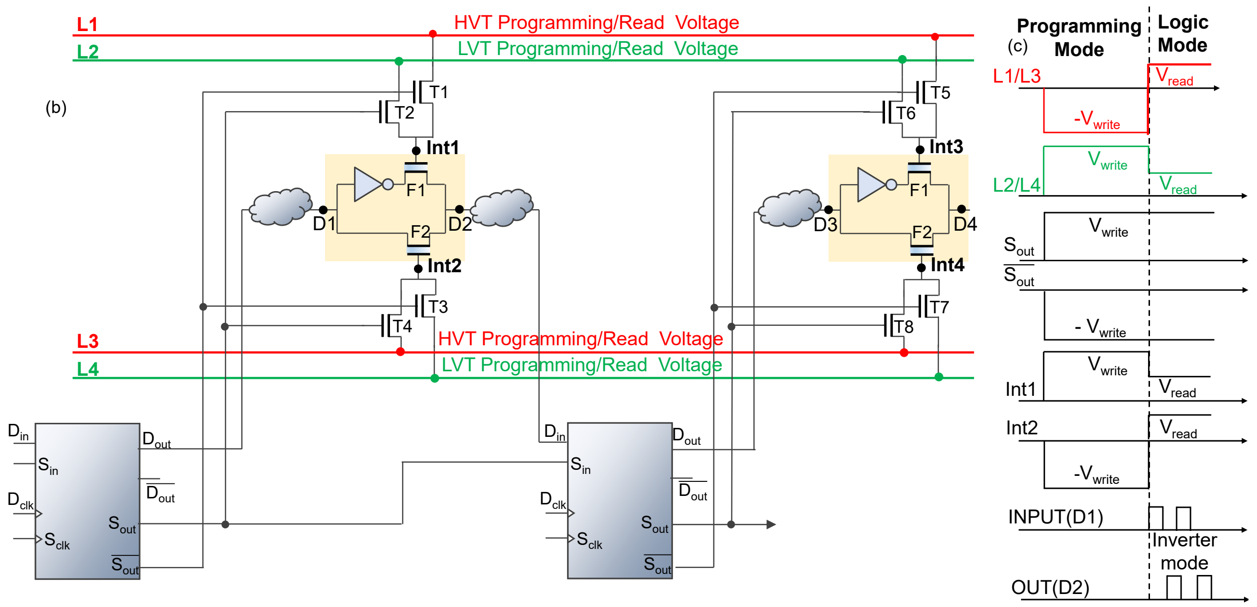

Figure S10: Circuit description of the proposed encryption key distribution with one step programming scheme. (a) The biasing for the peripheral scheme. (b) The peripheral circuit for programming. (c) Peripheral biasing waveforms in the programming and logic mode operation.

Programming mode and logic mode operating voltages are exerted on L1, L2, L3, L4 (

Fig. S10). In the programming mode, the two selector transistors in the top (T1 & T2) and the two selector transistors in the bottom (T3 & T4) act as switches to enable the transfer of programming write voltages to the encryption circuits. Complementary write voltages are given to the upper and lower part of encryption unit. $Scan\_out$ and $\overline{scan\_out}$ enable a pair of selector transistors and establishes a connection to FeFETs (F1, F2) either from L1 & L4 (buffer mode) or from L2 & L3(inverter mode). With this version of peripheral circuit, programming of both the FeFETs in the encryption circuit (F1 & F2) to complementary V_T state is done in one step by applying corresponding positive or negative write voltages as shown in Fig. S10(c). In logic mode, read voltages are exerted on L1, L2, L3 and L4 and these voltages get transferred to the gate of the ferroelectric FETs (F1, F2). Depending on the programmed state of the FeFETs the encryption block will either produce a buffered or an inverted version of input. The biasing for programming and logic modes are given in Fig. S10(a). Expected waveform on various metal lines during the operation is given in Fig. S10(c).
